# Autistic children who create imaginary companions: Evidence of social benefits

**DOI:** 10.1177/13623613221092195

**Published:** 2022-05-02

**Authors:** Paige E Davis, Jessica Slater, David Marshall, Diana L Robins

**Affiliations:** 1 York St John University, UK; 2 York University, UK; 3 Drexel University, USA

**Keywords:** autism spectrum disorders, imaginary companions, social understanding, theory of mind

## Abstract

Past research shows that autistic children can and do create imaginary companions (ICs), and that these ICs resemble those that neurotypical children create. Neurotypical children creating ICs have been found to have significantly more developed theory of mind (ToM) and social understanding among other enhanced social cognitive skills. The study set out to determine if this finding applies to autistic children. Parents of 124 (38 female) autistic children, ages from 5 to –12 years old, completed questionnaires evaluating communication, social understanding, and social skills. Children with ICs had significantly higher ToM and social skills scores regardless of their communication abilities. Findings suggest that there is a variability in ToM and social skills in autistic children in reference to an IC play profile. Results are discussed in terms of direction of causality and lab-based investigations.

**Lay abstract:**

Research on neurotypical children with imaginary friends has found that those with imaginary friends have better social skills and are more able to think about how other people’s minds work compared to children without imaginary friends. Research shows that some autistic children also create imaginary friends. This article is the first to look at whether or not autistic children with imaginary friends have stronger social skills and an improved ability to think about others’ minds than those without imaginary friends. We asked parents to report about their children aged 5 to 12. Finding almost half reported their child had an imaginary friend, a much larger number than previous research with younger children. Our findings also suggested that autistic children with imaginary friends were better able to understand others’ minds and had stronger social skills than their peers without imaginary friends. The children’s language ability did not influence this. The findings of this study add to the evidence that with respect to the creation imaginary friends and their potential benefits, the play profiles of autistic children are similar to the general population. It also provides more evidence that the understanding of others’ minds is not all or nothing in autism and gives reason for researchers to investigate whether the causes of these differences are the same or different for autistic children.

Imaginative play is of particular importance to children’s development, and appears to have a reciprocal relationship with broader social competence, in which children with impoverished play tend to also exhibit delayed social and developmental skills (Lillard, 2017; Stanley & Konstantareas, 2007). This may be because children who engage in pretend play may also be exposed to more opportunities to engage in social interaction (Hobson et al., 2009), learn to interpret social signals (Lillard, 2017), and think about how others’ minds work (Davis, 2020).

The play profile for autistic children may differ from their neurotypical peers. For example, they may be less likely to engage in pretend play, and when they engage in imaginative activities like drawing, ToM skills relate to visuospatial planning ability in autistic children, but not in a matched group of neurotypical children (Low et al., 2009). However, it has been found that ToM has been linked to the quality rather than the quantity of pretend play exhibited based on the Child-Initiated Pretend Play Assessment (Lin et al., 2017). Furthermore, it has been shown that even a five-week intervention that increases cognitive play skills and imagination via methods such as prompts, reflecting emotions, and modelling increases emotional understanding in autistic children (Doernberg et al., 2021). Children in the intervention group significantly improved describing their own emotions when explaining past experiences, whereas the control group did not.

The creation of an imaginary companion (IC) is one type of pretend play that has been found to be related to improved social competence and better social understanding in neurotypical children (Davis et al., 2014; Giménez-Dasí et al., 2016; Smith, 2019; Taylor & Carlson, 1997). Specifically, IC creation in children has been linked to better referential communication skills (Roby & Kidd, 2008), increased focus on mental states in friends (Davis et al., 2014), and superior ToM and emotional understanding (Giménez-Dasí et al., 2016; Taylor & Carlson, 1997). Children with ICs were first found to excel at ToM by Taylor and Carlson (1997). They examined interpretive diversity, where a child was shown a picture and then a section was covered and the child would be asked to determine if someone who had never seen the image could make out what it was from only the section that was revealed. They also looked at appearance reality, having the child distinguish between real and apparent identity and colour as well as false belief using a version of the smarties task (Perner et al., 1987).

Giménez-Dasí and colleagues (2016) replicated and expounded upon the finding that children with an IC have significantly better ToM than those who do not create an IC, also looking at social competence and showing IC children show the same proclivity. ToM findings in neurotypical populations have not been replicated in some studies (Davis et al., 2011; Fernyhough et al., 2007), suggesting that IC play may be related to only certain aspects of ToM development involving appearance reality, false belief and interpretive diversity rather than stream of consciousness, or intention (Davis, 2020; Taylor & Carlson, 1997).

Social communication, ToM and social understanding are all areas in which autistic children have been shown to have differences (American Psychiatric Association (APA), 2013; Baron-Cohen, 1987). These differences, when paired with pretend play skills that do not resemble the typical presentation, might suggest less inclination to create ICs. However, it has been found that 16% of parents of autistic children report their child playing with an IC. Furthermore, parents of autistic children can answer in depth questions about their child’s IC (Calver, 2009; Davis et al., 2018). Davis and colleagues (2018) found that although significantly fewer autistic children create ICs, and their ICs are more likely to be personified dolls or toys rather than completely invisible, the ICs that they do create did not differ in social attributions children afforded them, or whether the children reported their function as social or comfort, compared to neurotypical peers. They also did not differ in the gender ascribed to them by the child. Thus, when parent reports are compared, many similarities in IC form and function arose between autistic children and neurotypical children.

Sex differences are sometimes found in children with ICs, as more females have been found to create them (Armah & Landers-Potts, 2021; Taylor, 1999), although Davis and colleagues (2018) did not find sex differences among autistic participants. One potential reason for this gender finding in neurotypical population is that parents are more accepting of female pretend play (Gleason, 2005). Giménez-Dasí and colleagues (2016) found that girls with ICs were significantly better at emotional understanding than boys with ICs, in addition to the overall IC effect on ToM. It has also been suggested that the expression of autistic features may differ across genders (Lai et al., 2017; Rivet & Matson, 2011); these findings may be a further by-product of this difference in expression.

One of the reasons why ICs are thought to improve children’s social skills and ToM understanding is because the creation of another mind gives them the opportunity to think about how other minds work (Davis, 2020). It also gives the child a chance to practice social interactions in the absence of a real individual (Davis, 2020; Gleason, 2017). This could be particularly useful to an autistic child who may not have as many chances to interact socially (Davis et al., 2014; Taylor, 1999; Taylor & Carlson, 1997), due in part to misinterpretation of autistic behaviour as indicating lack of social motivation (Jaswal & Akhtar, 2019).

Given that autistic children have been found to create ICs and their ICs seem to be qualitatively similar to neurotypical children’s creations, it follows that those autistic children who create ICs may also exhibit higher ToM and social understanding scores. The aim of this preliminary investigation was to determine whether parents of autistic children will report IC creators as having more developed ToM and social skills than the children who do not have an IC. We designed this study with similar methods to both Taylor and Carlson (1997) and Giménez-Dasí and colleagues (2016), thus communication ability will be partialed out and the IC category will incorporate both invisible ICs and personified dolls and toys.

We hypothesised that parents who report that their children have ICs will also report significantly higher scores on (1) ToM and (2) social skills measures than those that report no ICs, regardless of communication ability. Girls will also outperform boys on the social skills measures mirroring Giménez-Dasí and colleagues’ (2016) sex differences.

## Method

### Participants

Participants were 124 parents reporting on their autistic children. Children were aged between 5 and 12 years (*M* = 8.34 years, *SD* = 2.14) and 69.4% (86) were male.

### Materials and procedure

Parents were recruited through Facebook support groups for parents of autistic children, and through a participant sourcing database called Prolific which has been found to source diverse populations and produce high quality data (Peer et al., 2017). The 49 participants from the Facebook groups were directed to the online Qualtrics questionnaire. They were able to access the questionnaire through their phone, tablet, or computer. There were originally 1,000 participants sourced from Prolific who took a pre-test where they had to indicate age and child’s autism diagnostic status. There were 80 participants that matched the criteria, and they were invited to participate in the main study and grouped into groups of 20. Only when the first 15–20 had finished the questionnaire would another 20 be asked to complete. This is because the participant is paid depending on how long the participant takes to complete the survey and the researchers had a set budget. There were 75 participants that were sourced from this site. The questionnaire was structured so that parents were asked about demographics, and IC status, followed by communication, ToM and social skills. It took parents anywhere from 5 to 30 min to fill out. Prolific participants were asked if they would be interested in participating in face-to-face interviews in the future before the questionnaire was compete. This protocol was approved by the university ethics committee.

### Measures

#### Demographic questions

After completing the consent questions which included age and diagnostic screeners, parents were asked the age and sex of their child. This was followed by three questions about their child’s IC status. ICs were explained, ‘*An imaginary friend can be invisible or a doll or a toy which your child has given a personality to and played with for at least 3* *months’*, and asked whether their child had an IC. If so, they were asked at what age their child had created the IC and why they thought the companion was created.

#### Children’s Communication Checklist - 2 (CCC-2)

The CCC-2 is a commonly used assessment employing parent or teacher report to look at language and pragmatic skills (Bishop, 2003; also see Bishop, 2006; Parsons et al., 2019). It consists of 10 scales with seven items each, five of which describe difficulties and two strengths. Because we were only interested in general language ability in order to control for this factor in terms of children’s ToM and social skills, only scales 1–4 were used. These scales measure (1) speech, (2) syntax, (3) semiotics and (4) coherence. Parents were asked to rate their child on a Likert-type scale from 0–3 indicating how often their child does what the statements say. Zero indicated less than once a week or never, one represented at least once a week, but not every day, two was once or twice a day, and three several times a day or always. Parents were asked questions such as, ‘*Produces utterances that sound babyish because they are just 2 or 3 words long, such as ‘me got ball’ instead of ‘I’ve got a ball’* or *‘give dolly’ instead of ‘give me the dolly’* or ‘*Explains a past event (e.g. what s/he did at school, or what happened at a football game) clearly’.* After reverse questions were calculated, the four scales were summed and sum scores could range from 0–84 where a lower score indicates more developed communication ability; raw scores were used to partial out communication abilities. Reliability analysis incorporating all questions from the four scales was excellent with an alpha coefficient at .93.

#### The Children’s Social Understanding Scale-short (CSUS)

The CSUS short was used to examine differences in parent report of ToM and social understanding (Tahiroglu et al., 2014). This assessment was created to mirror the content of ToM tasks given in the lab and has been found to be a reliable and valid measure of ToM in children (Tahiroglu et al., 2014). The CSUS short consists of six scales with seven questions per scale. Parents are asked to rate their child on a 4-point Likert-type scale with an additional option of ‘don’t know’. This study used the belief, perception and emotion scales as these most closely mirror the type of ToM measured in Taylor and Carlson (1997) and Giménez-Dasí and colleagues (2016) IC, ToM, and social understanding work. One false belief question reads, *‘Talks about people’s mistaken beliefs (e.g. He thought it was a dog, but it was really a cat’”; I thought mommy was coming but it was really daddy’)’.* The perception scale has questions such as *‘Talks about the way things look and how they really are (e.g. ‘It looks like a snake, but it’s really a lizard’)’.* And the emotion scale has questions like, *‘Tries to understand the emotions of other people (e.g. wants to know why you are crying)’.* After answers were reversed and summed, scores can range from 21–84. A higher score indicates more developed ToM knowledge. Scores were not included where parents indicated that they did not know about their child’s behaviour. Reliability analysis for total score was good with an alpha coefficient at 0.87, as well as good alpha coefficients for subscores on belief at 0.81 and emotion at 0.80, but poor for the perception subscale at 0.49.

#### The TRIAD Social Skills Assessment Second Edition (TSSA)

The TSSA was used to assess a child’s social skills both at home and in a community setting based on parent observations (Stone et al., 2010). The parents completed part of the social skills survey section of the TSSA explaining how many friends their child has, how interested they are in spending time with peers and making friends. These three questions were summed creating a friends’ subscale, and used alone looking at IC and no IC group differences. Parents also completed the social skills rating form sections using a Likert-type scale to rate their child’s abilities ranging from 1 – not very well to 4 – very well. The sections included were (1) initiating interactions, with questions such as, *‘how well does your child start conversation with others?’* (2) responding to initiations, *‘how well does your child respond in a friendly manner when he or she is greeted by others?’* (3) Maintaining interactions, *‘how well does your child stay on topic during conversations?’* and (4) six follow up questions spanning all sections. We did not ask parents to answer the questions on affective understanding and perspective taking as we had already asked them about this using the CSUS. Scores were summed for sections and totalled for the whole rating form. The range for the sum score was 35–140 where a higher score indicated more developed ability. Reliability analysis was excellent with an overall alpha coefficient of 0.97 and excellent alpha coefficients for all subscales; Initiating interactions at 0.92, responding to initiations at 0.92, maintaining interactions at 0.92 and follow up questions at 0.92 with the exception of a good alpha coefficient for the making friends category at 0.86.

### Planned analysis

Main data analysis planned to use the Statistical Package for the Social Sciences to determine if there would be differences in how parents score their children’s ToM and social skills between the IC and no IC groups of children regardless of their communication ability. This was done through looking at whether there were (IC x no IC) group differences in CCC-2, CSUS, and TSSA scores using an analysis of variance (ANOVA). Age, sex and raw CCC-2 scores were investigated to determine if they were related to IC status and considered in the CSUS and TSSA analyses if they were. A MANCOVA was used to look into CSUS and TSSA sub scores partialing out CCC-2 scores.

There was no community involvement in the reported study other than the advice and permissions granted by the gatekeepers for the Facebook groups who helped promote the study.

## Results

### Descriptive statistics and preliminary analyses

Based on the parent report, 49.2% of autistic children were reported to have created an IC between 1 and 11 years old (*M* = 5.30, *SD* = 1.95). For means and standard deviations for standardised measures for each IC group, see Table 1. The TSSA questions relating to friendships, where means for the IC group were higher (*M* = 2.18, *SD* = 1.60) than the no IC group (*M* = 1.86, *SD* = 1.67), showed that IC status did not relate to how many close real friends a child had *t* (1,117) = −1.07, *p* = .289, but children with ICs were significantly more likely to be interested in making friends and spending time with them (*M* = 5.97, *SD* = 1.88) than those without (*M* = 5.08, *SD* = 2.56), *t* (1,108) = −2.16, *p* = .033. η^2^ = .041. Current child age was not related to a parent reporting that their child had created an IC *t* (1,122) = −1.32, *p* = .188, so this variable was not considered in further analyses. However, child sex was related to IC status *X*^2^(1, *N* = 124) = 4.28, *p* = .039; parents of girls (63.2%) were more likely to report their child having created an IC than parents of boys (43%). Finally, there are significant correlations between the CCC-2 and both the CSUS as well as the TSSA; see Table 2.

**Table 1.** Mean (standard deviation) scores as a function of imaginary companion status.

	Group	N	Range*	Mean (*SD*)
Current Age (Years)	IC	61	5–12	8.08 (1.94)
	No IC	63	5–12	8.59 (2.30)
	Total	124	5–12	8.34 (2.14)
Communication Sum Score (CCC-2)	IC	54	22–79	44.15 (13.99)
	No IC	55	23–97	47.84 (17.48)
	Total	109	22–97	46.01 (15.88)
Theory of Mind Sum Score (CSUS)	IC	47	44–80	65.62 (8.96)
	No IC	46	37–82	55.96 (10.62)
	Total	93	37–82	60.84 (10.90)
Social Skills Sum Score (TSSA)	IC	59	48–126	88.34 (18.18)
	NIC	60	36–135	71.98 (24.59)
	Total	119	36–135	80.09 (23.07)

* *Note* All ages are reported in years. Test ranges are raw scores.

**Table 2.** Correlation table for communication, theory of mind, and social skills scores, and age.

Variable	*n*	*M*	*SD*	1	2	3	4
1. CSUS Sum Score	93	8.34	2.14	__			
2. TSSA Sum Score	119	80.09	23.07	.631**	__		
3. CCC-2 Sum Score	109	46.01	15.88	−.654**	−.406**	__	
4. Child’s Age (Years)	124	8.34	2.14	.091	−.042	−.227*	__

** Correlation is significant at the 0.01 level (2-tailed).

* Correlation is significant at the 0.05 level (2-tailed).

### IC status and parent report of communication skills

An ANOVA was run looking at IC status and parent report of communication skills via CCC-2 and no relation was found, *F* (1,108) = 1.50, *p* = .223, ηp^2^ = .014. Sex was also considered in the ANOVA, and there was no relationship between sex and CCC-2 score, *F* (1,108) = 1.66, *p* = .200, ηp^2^ = .016. There was also no interaction effect found, *F* (1,108) = .015, *p* = .902, ηp^2^ < .001. Even though there were no differences between the IC groups, CCC-2 scores were still added as covariates into the rest of the analyses as they have been found to link to ToM in past research (Astington & Baird, 2005).

### IC status and theory of mind

An ANCOVA was then run to determine if there was a relationship between IC status and parent report of ToM via CSUS scores, where sex was also considered as a fixed factor and communication score was a covariate. Parents of children with ICs scored their children significantly higher on the ToM measure than those with no IC, *F* (1,32) = 10.77, *p* < .003 ηp^2^ = .285; there was a large effect size for this result. No sex differences were found for the ToM scores, *F* (1,32) = .01, *p* = .933, ηp^2^ < .001. There were no interactions between IC status and sex, *F* (1,32) = 2.31, *p* = .141, ηp^2^ = .079.

In order to look at whether there were more fine-grained differences in components of ToM, namely belief, perception, and emotion, a MANCOVA was run with IC status and sex as fixed factors and CCC-2 scores as a covariate. The CCC-2 was found to make a significant independent contribution to the belief scale. ICs also were found to make an independent significant contribution over and above the CCC-2 contribution. Parents of children with ICs were significantly more likely than parents of children without ICs to report more advanced ToM in two of the three areas: belief and emotion, but not perception. The belief and emotion results had large effect sizes. There were no sex differences or interaction effects. See Table 3 for MANCOVA results.

**Table 3.** MANCOVA results for children’s social understanding scale subscales.

Fixed Factors	Dependant Variables	F	Sig.	ηp^2^
	Belief	12.47	.001	.287
CCC-2	Perception	0.61	.442	.019
	Emotion	1.70	.202	.052
IC	Belief	7.02	.013	.185
	Perception	0.09	.763	.003
	Emotion	8.33	.007	.212
Sex	Belief	0.14	.711	.004
	Perception	0.07	.793	.002
	Emotion	0.56	.459	.018
IC * Sex	Belief	0.62	.439	.019
	Perception	0.20	.659	.006
	Emotion	0.46	.504	.015

Significant correlations were found between all three areas of ToM: belief with perception *r*(107) = .396, *p* = .001; belief with emotion *r*(104) = .690, *p* = .001 and emotion with perception *r*(107) = .355, *p* = .001.

### IC status and social skills

Finally, a third ANCOVA was run to determine whether the IC status related to parent report of social skills with sex as a fixed factor and CCC-2 score as a covariate. Having an IC related to parent rating of significantly better social skills through the TSSA questionnaire *F* (1,37) = 24.81, *p* < .001, ηp^2^ = .429, with a large effect size for this result. Sex was not related to social skills *F* (1,37) = .55, *p* = .463, ηp^2^ = .016, and there was no interaction between sex and IC status *F* (1,37) = .194, *p* = .663, ηp^2^ = .006.

In order to look at the differences in TSSA scales, a MANCOVA was run with making friends, initiating interactions, responding to interactions, maintaining interactions and follow up overview scales as dependent variables, and IC status and sex as fixed factors and CCC-2 score as a covariate. Again, the CCC-2 was found to make independent significant contributions to all scales in the TSSA subscales. Results also indicated that IC status made independent significant contributions to the scores over and above the contributions of the CCC-2. Parents reported significantly higher scores for children with ICs for every sub scale: making friends, initiating interactions, responding to interactions and maintaining interactions. All significant results had large effect sizes. Sex was not related to any of the scales, and there were no interaction effects with between IC status and sex. Results for the TSSA subscale MANCOVA can be found in Table 4. Significant correlations were found between all TSSA subscore variables. See Table 5.

**Table 4.** MANCOVA results for the TRIAD social skills assessment subscales.

Fixed Factors	Dependant Variables*	F	Sig.	ηp^2^
	Friends	9.54	.004	.224
CCC-2	Initiating	19.55	< .001	.372
	Responding	8.02	.008	.196
	Maintaining	11.86	.002	.264
	Overview	14.60	< .001	.307
IC	Friends	15.53	< .001	.320
	Initiating	22.92	< .001	.410
	Responding	5.07	.031	.133
	Maintaining	19.50	< .001	.371
	Overview	20.113	< .001	.379
Gender	Friends	0.00	.976	.000
	Initiating	0.33	.570	.010
	Responding	0.02	.885	.001
	Maintaining	0.73	.399	.022
	Overview	2.44	.128	.069
IC * Gender	Friends	1.14	.844	.001
	Initiating	2.10	.651	.006
	Responding	1.95	.642	.007
	Maintaining	4.65	.506	.014
	Overview	0.23	.632	.007

* *Note* Subscales full names are interest in making friends (Friends), initiating social interactions (Initiating), responding to social interactions (Responding), maintaining social interactions (Maintaining).

**Table 5.** Correlation table for TSSA sub score categories.

Variable	*n*	*M*	*SD*	1	2	3	4	5
1. Making friends	112	9.11	4.83	__				
2. Initiating interaction	120	25.09	8.37	.657*	__			
3. Responding to interaction	119	13.18	3.95	.609*	.770*	__		
4. Maintaining interaction	120	22.70	7.27	.593*	.711*	.578*	__	
5. Overview questions	120	11.30	3.60	.599*	.748*	.602*	.840*	__

* Correlation is significant at the 0.01 level (2-tailed).

### Communication, ToM, social skills and age

A Pearson’s bivariate correlation was run to look at relations between the CCC-2, CSUS, TSSA and age. The CCC-2 was related to the TSSA, *r*(106) = −.41, *p* *<* .001 and CSUS, *r*(84) = −.65, *p* *<* .001. The TSSA was related to the CSUS, *r*(92) = .631, *p* *<* .001, whereas child age was only related to parent report of communication through the CCC-2, *r*(109) = −.23, *p* = .018. See Table 5 for correlations.

## Discussion

The aim of this investigatory study was to determine, based upon retrospective parent report, whether or not autistic children who have created an IC show higher social emotional and ToM scores than those who had not created an IC, similar to prior research on neurotypical children with ICs. Almost half of the 124 parents reported that their child had created an IC and these parents rated their children significantly higher than those who did not create ICs on both ToM and social skills inventories regardless of communication ability. While communication was shown to make significant independent contributions to these scales, IC status also made independent significant contributions to both ToM and social skills above and beyond the communication scale. These significant results had large effect sizes. Parents of boys were less likely to report their child as having created an IC than parents of girls, and those reporting that their child had created an IC also reported their child as having significantly more interest in making and spending time with real friends, although IC status did not relate to the number of real friends the child already had made.

Almost half of the parents surveyed for this study reported that their child had created an IC. This play profile was unexpected, as Davis and colleagues (2018) had found only 16% of the parents surveyed had reported their child had an IC. However, this study used a much older population than the prior work. There were no age differences between the IC and no IC groups, but parents of girls were more likely to report their child having an IC than parents of boys. It has been suggested by Gleason (2005) that girls are more likely to create ICs, and it has been theorised that this results from parents being more likely to encourage girl IC play, but other studies have failed to find this association (Armah & Landers-Potts, 2021).

In terms of friendship, children with ICs were not reported as having closer real-life friends. However they were significantly more interested in making new friends and spending time with peers than children with no IC. This maps on to the theory that children with ICs create them to compensate for real life friends with whom they experience challenges interacting (Gleason, 2017; Hoff, 2004). This could be an example of a way in which autistic children express social interest (Jaswal & Akhtar, 2019).

This study replicated the results of both Taylor and Carlson’s (1997) and Giménez-Dasí et al.’s (2016) studies in autistic children, although the current study relied on parents’ report rather than direct testing of children. Children with ICs were reported to be significantly more developed in their ToM ability as well as their social skills regardless of their aptitude for communication. When sub scales were investigated, the perception subscale did not show any between-group differences, whereas IC children scored significantly higher on emotion and belief subscales than those without ICs. The lack of significant difference in perception may be related to poor internal consistency of the perception subscale. Alternatively, it could be due to the children with ICs excelling in certain domains of ToM and not others (Davis, 2020; Taylor & Carlson, 1997) which would further refute the idea that ToM is dichotomous, and that one either has or does not have ToM and that autistic children lack a ToM altogether (Baron-Cohen et al., 1985). All subscales for the social skills inventory showed IC children scored significantly higher than children without ICs. No sex differences were found for either ToM or social skills measures. This refutes Giménez-Dasí et al.’s (2016) findings that girls with ICs had better emotion understanding than boys with ICs and all children with no IC.

It is important to note that the direction of causality has not been established for the relationship between ICs, ToM and social skills. Longitudinal research supports both directions; children already excelling in these socio-cognitive areas are more likely to create ICs (Moriguchi et al., 2016; Motoshima et al., 2014) and also that playing with ICs and shaping another’s mind can self-scaffold a child, helping them to think about perspectives, emotions and how the mind works, thus helping them learn about those minds through their mental representation of the IC (Davis, 2020; Gleason, 2017; Lillard & Kavanaugh, 2014). A third possibility would be that ToM, social skills and ICs are caused by another variable that is not yet identified. This would mean that all three areas were impacted by this unidentified variable, thus direction of causality for this relationship will be important to examine in future research. Investigating these causal models in autistic children is important given that relationships among these variables of interest may differ compared to neurotypical children (i.e. Karmiloff-Smith, 1998).

An important limitation of this study was that there was sole reliance on parent report. This study, like Davis et al. (2018) was based only on retrospective parent report which comes with some inherent issues. The retrospective nature does not take into account that ICs can be forgotten by parents (Davis et al., 2019) and the parent report means that the study loses the child’s voice along with ecological validity. One issue specific to this study was that parents completed the questionnaire more quickly than anticipated. This could have been because they were not reading questions carefully. COVID-19 was one factor in this decision to restrict data collection solely to parent report, although prior work indicates that parents are good at reporting on their child’s inner lives and can provide greater ecological validity observing them over time in different contexts (Gleason, 2004; Tahiroglu et al., 2014). Although there are arguments that parents of autistic children might be less likely to know that their child has an IC (Davis et al., 2018). It was preferable to investigate via survey first to determine if this was even an effect and to later match children with and without ICs on the same communication levels and see them in a lab context.

Another limitation was in the method of delivery. Having an online questionnaire for parents meant that it needed to be as short as possible so that participants did not lose interest and their time was not wasted. This meant that although the participants had less burden on them in terms of answering questions, the questionnaire sacrificed detail. For example, not all of the subscales in the CCC-2, CSUS or TSS were used. Furthermore, there was not as much specificity as there could have been when it came to IC status and autism diagnostics. There were no questions about type of IC (invisible or personified object like a doll or toy) or specific diagnostics (i.e. age of diagnosis, type of autism). Future research should include these assessments in their entirety. Furthermore, including IC type would be interesting for future research to determine if type matters for social skills or ToM. In the original Taylor and Carlson (1997) article, type of IC was not related to ToM skills leading them to conclude that the child’s creation of another social being with a mind to interact with would use the same underlying mechanisms (Davis et al., 2014; Taylor & Carlson, 1997), however this may be different when looking at autistic children.

Finally, looking at autism-specific factors like camouflaging or being in an autism-specific environment could also be variables that are impacting autistic children that do and do not create ICs (Davis & Crompton, 2021). Autism-specific environments have been shown to enrich both school and life experiences as well as decrease camouflaging and feelings of social isolation (Davis & Crompton, 2021). Looking at autistic children’s IC play profiles who do and do not interact with other autistic children could be another avenue to explore in social skills and imagination.

Broadly, these results suggest that both the ToM and social skills advantages seen in neurotypical children are also seen in parents’ reports of autistic children. Results also suggest that ToM is not a unitary construct in autistic children, and that different play profiles seem to be related to variability in social and ToM scores. This adds to the argument that autistic children have ICs that are conceptualised similarly to those of neurotypical children. If this finding is found to be consistent, it could have important ramifications for future imagination work with autistic children.
